# Improvement in Adherance to Anesthesia Preoperative Appointment With Telemedicine: A Retrospective Analysis

**DOI:** 10.7759/cureus.60805

**Published:** 2024-05-21

**Authors:** Danny Q Le, Brittany N Burton, Christian J Tejeda, Laleh Jalilian, Nirav Kamdar

**Affiliations:** 1 Anesthesiology, University of California Los Angeles David Geffen School of Medicine, Los Angeles, USA; 2 Anesthesiology and Perioperative Medicine, University of California Los Angeles David Geffen School of Medicine, Los Angeles, USA; 3 Quality Improvement and Clinical Operations, Huntington Hospital, Pasadena, USA

**Keywords:** appointment adherence, anesthesia care, covid-19, preoperative medicine, telemedicine (tm)

## Abstract

Background: Amidst the coronavirus disease 2019 (COVID-19) pandemic, the sudden demand for virtual medical visits drove the expansion of telemedicine across all medical specialties. Current literature demonstrates limited knowledge of the impact of telehealth on appointment adherence, particularly in preoperative anesthesia evaluations. This study aims to describe the impact of telemedicine-based anesthesia evaluation and its effects on appointment completion.

Methods: This was a retrospective, non-randomized, cohort study of adult patients at the University of California, Los Angeles, United States, who received preoperative anesthesia evaluations by telemedicine or in-person in an academic medical center. From January to September 2021, we evaluated telemedicine and in-person appointment completion in patients scheduled for surgery. The primary outcome was the incidence of appointment completion. The secondary outcomes included appointment no-shows and cancellations.

Results: Of 1332 patients included in this study, 956 patients received telehealth visits while 376 patients received in-person preoperative anesthesia evaluations. Compared to the in-person group, the telemedicine group had more appointment completions (81.38% vs 76.60%), fewer cancellations (12.55% vs 19.41%), and no statistical difference in appointment no-shows (6.07% vs 3.99%). Compared to the in-person group, patients who received telemedicine evaluations were younger (55.81 ± 18.38 vs 65.97 ± 15.19), less likely Native American and Alaska Native (0.31% vs 1.60%), more likely of Hispanic or Latino ethnicity (16.63% vs 12.23%), required less interpreter services (4.18% vs 9.31%), had more private insurance coverage (53.45% vs 37.50%) and less Medicare coverage (37.03% vs 50.53%).

Conclusions: This study demonstrates that telemedicine can improve preoperative anesthesia appointment completion and decrease appointment cancellations. We also demonstrate potential shortcomings of telemedicine in serving patients who are older, require interpreter services, or are non-privately insured. These inequities highlight potential avenues to increase equity and access to telemedicine.

## Introduction

Amidst the coronavirus disease 2019 (COVID-19) pandemic, medical specialties expanded telemedicine services to meet the demand for virtual medical visits despite its historical resistance [1-6]. The field of anesthesiology was no exception; the American Society of Anesthesiologists (ASA) and the American College of Surgeons (ACS) officially called for the utilization of teleconsultations for preoperative evaluations to maintain medical services while limiting disease transmission [7,8]. Additionally, anesthesiologists increasingly utilized telemedicine for pain management in both rural and urban healthcare settings [9]. The in-person preoperative anesthesia consultation serves multiple purposes including patient education, optimization of comorbid conditions, and perioperative risk reduction [10-14].

Historical resistance to telemedicine has included concerns about the development of the patient-provider relationship and lack of physical examination. Although these concerns are valid, they were mostly theoretical. Studies showed high satisfaction in the degree of trust with physicians during the pandemic while using telemedicine methods [15]. Surgical plans developed via telemedicine visits remain unchanged after in-person examinations [16]. In addition, virtual airway evaluations, critical to the development of anesthesia intraoperative plans, have been found to have strong agreement with in-person airway evaluations [17]. Current medicine practice places a strong focus on objective skills and evaluations but the virtual visit can still provide much physical objective data, particularly visual in addition to objective studies such as echocardiograms and chest x-rays, which can be obtained after subjective histories and review of systems. 

Although considered an imperfect modality, telemedicine has increased access to medical care for patients and reduced consultation time, distance traveled, emission of environmental pollutants, and related costs while maintaining high patient satisfaction [4,8,18-21]. The current literature also shows that telemedicine provides patients with time and cost-saving benefits while avoiding further day-of-surgery delays [8,22]. Preoperative anesthetic telemedicine is a potentially cost-effective and convenient modality for overhead reduction, digitally interfacing with patients across greater distances, and innovation within the specialty [22,23]. Researchers have found that both providers and patients expressed overall satisfaction with preoperative telemedicine visits [10,24,25].

Telemedicine can be utilized to develop anesthetic plans and evaluate the severity and progression of the principal disease, other comorbid conditions, and the urgency of the procedure [13]. It can also be employed for timely screening and triaging of patients with suspected or established COVID-19 [10,13]. While telemedicine has been demonstrated to improve appointment adherence in nonsurgical and surgical specialties, there is limited knowledge of its effects on appointment adherence for preoperative anesthesia appointments [21,26]. Barriers to telemedicine implementation include the scarcity of standardized protocols, strict federal and state regulations, upfront costs, and education of healthcare personnel [13,24]. Furthermore, there is limited information on disparities in access to telehealth for preoperative anesthesia evaluations. Current studies suggest conflicting evidence on the social determinants of telehealth equity.

As such, in this study, we aim to compare the impact of telemedicine versus in-person preoperative anesthesia evaluations on appointment completion at a single, urban, academic medical center. Secondarily, we evaluated appointment cancellations, no-shows, group differences in patient demographics and socioeconomic factors, and reported reasons for appointment cancellations. We hypothesized that preoperative anesthesia evaluations via telemedicine compared to in-person would have higher appointment completion. 

This article was previously presented as a meeting abstract at the 2022 International Anesthesia Research Society (IARS) Conference on March 18, 2022.

## Materials and methods

The University of California, Los Angeles (UCLA) Health telemedicine preoperative anesthesia evaluation was initiated on August 1, 2017, within our division’s Pre-operative Evaluation and Planning Center (PEPC). This was a retrospective, non-randomized cohort study of all adult patients who received preoperative anesthesia evaluations by telemedicine or in-person from January to September 2021 at UCLA Health. The study was approved by the Institutional Review Board of UCLA (approval number: 19-000554 dated March 15, 2020), and patient consent was waived. 

The data collected included appointment completion, cancellation, no show, PEPC secretary-reported reasons for patient cancellation, and patient demographics. The primary outcome was the incidence of appointment completion. The secondary outcomes included appointment no-shows and cancellations. Patient demographic characteristics including sex, age, ASA physical status class, race, ethnicity, primary language, interpreter service requested, patient travel distance to clinic, and insurance payor were also evaluated. The primary outcomes and subgroup analysis were established a priori at the initiation of the study. The PEPC secretary reported reasons for cancellations were also reviewed and categorized into thematic groups by two physicians. This article adheres to the applicable Strengthening the Reporting of Observational Studies in Epidemiology (STROBE) guidelines.

Statistical analyses

Patient characteristics and study variables were summarized between the telemedicine and in-person cohorts using mean (standard deviation) or frequency (%) unless otherwise noted and formally compared using the independent samples t-test, Pearson’s chi-square, and Fischer’s exact test, as appropriate. The sample size was justified by resource constraints. Data analysis was performed using JMP Pro v.15.0.0 statistical software (SAS Institute Inc., Cary, North Carolina, United States) and p-values <0.05 were considered statistically significant.

## Results

Demographics

A total of 1332 preoperative anesthesia evaluations were conducted during the study period. Of these, 956 patients received telehealth visits while 376 patients received in-person preoperative anesthesia evaluations. On average, the population who used the UCLA PEPC telemedicine program were predominantly younger, less likely of American Indian race, more likely of Hispanic or Latino ethnicity, used English as a primary language, required less interpreter services, and were more likely privately insured and less likely insured by Medicare. Patient demographic characteristics are presented in Table 1.

**Table 1:** Demographic characteristics of study participants Data presented as n (%) or mean (SD). *p-values < 0.05 were considered significant. ASA: American Society of Anesthesiologists

Variables	Telemedicine Evaluation, n (%)	In-Person Clinic Evaluation, n (%)	P-value
Sex			0.0435*
Male	309 (32.32%)	148 (39.36%)	
Female	646 (67.57%)	228 (60.64%)	
Age (years)	55.81 (18.38)	65.97 (15.19)	<0.001*
ASA			0.3804
I	12 (1.26%)	3 (0.80%)	
II	302 (31.59%)	110 (29.26%)	
III	598 (62.55%)	238 (63.30%)	
IV	44 (4.60%)	25 (6.65%)	
Race			
Native American	3 (0.31%)	6 (1.60%)	0.0102*
Asian	72 (7.53%)	30 (7.98%)	0.7823
Black	84 (8.79%)	31 (8.24%)	0.7513
Declined	29 (3.03%)	9 (2.39%)	0.5278
Other	172 (17.99%)	58 (15.43%)	0.2647
White or Caucasian	588 (61.51%)	236 (62.77%)	0.6701
Pacific Islander	1 (0.10%)	2 (0.53%)	0.1387
Unknown	7 (0.73%)	4 (1.06%)	0.5472
Ethnicity			
Hispanic or Latino	159 (16.63%)	46 (12.23%)	0.0453*
Not Hispanic or Latino	742 (77.62%)	309 (82.18%)	0.066
Declined	9 (2.39%)	25 (2.62%)	0.8176
Unknown	30 (3.14%)	12 (3.19%)	0.9600
English as Primary Language	903 (94.46%)	324 (86.17%)	<0.001*
Interpreter Required	40 (4.18%)	35 (9.31%)	0.0003*
Distance to Clinic	99.84 (343.09)	91.55 (503.21)	0.7693
Insurance Coverage			
Medicaid	57 (5.96%)	30 (7.98%)	0.18
Medicare	354 (37.03%)	190 (50.53%)	<0.0001*
Private	511 (53.45%)	141 (37.50%)	<0.0001*
Self-Pay	1 (0.10%)	0 (0%)	0.5304
Other	8 (0.84%)	6 (1.60%)	0.2215
Unknown	25 (2.62%)	9 (2.39%)	0.8176

Appointment completion

**Figure 1:**
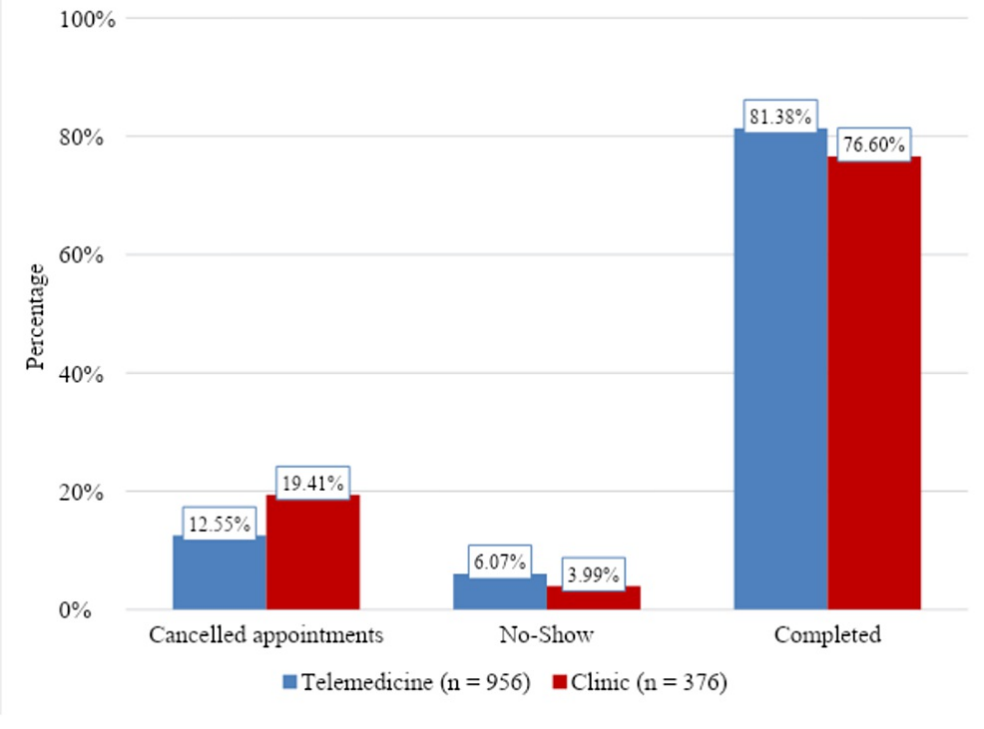
Comparison of cancellation, no-show, and completion of appointments between patients who received telemedicine or in-person (clinic) preoperative evaluation appointments.

Out of 956 patients evaluated by telemedicine, 778 (81.38%) patients completed their scheduled preoperative anesthesia evaluations (Figure 1). Out of 376 patients evaluated in person, 288 (76.60%) completed their scheduled preoperative anesthesia evaluations. Compared to the in-person group, the telemedicine group had significantly more appointment completions (81.38% vs 76.60%, p = 0.0493).

Appointment cancellation

Out of 956 patients evaluated by telemedicine, 120 (12.55%) patients canceled their scheduled preoperative anesthesia evaluations (Table 2). Out of 376 patients evaluated in person in the clinic, 73 (19.41%) canceled their scheduled preoperative anesthesia evaluations. Compared to the in-person group, the telemedicine group had significantly fewer appointment cancellations (12.55% vs 19.41%, p = 0.0029).

**Table 2:** Comparison of cancellation, no-show, and completion of appointments between patients who received telemedicine or in-person preoperative evaluation appointments. *Statistically significant difference between groups

	Telemedicine Evaluation	In-Person Clinic Evaluation	P-Value
Total Patients	956	376	0.0029*
Canceled, n (%)	120 (12.55%)	73 (19.41%)	0.0014*
No-Show, n (%)	58 (6.07%)	15 (3.99%)	0.1337
Completed, n (%)	778 (81.38%)	288 (76.60%)	0.0493*

Appointment no show

Out of 956 patients evaluated by telemedicine, 58 (6.07%) patients did not show up to their scheduled preoperative anesthesia evaluations. Out of 376 patients evaluated in person, 15 (3.99%) patients did not show up to their scheduled preoperative anesthesia evaluations (Table 2). Compared to the in-person group, no-show rates in the telemedicine cohort were not significantly different (6.07% vs 3.99%, p = 0.1337).

Reasons for appointment cancellation

There were 73 patient cancellations amongst the entire cohort of patients who received telemedicine or in-person preoperative anesthesia evaluations. The top four reasons for cancellation included patient request or reason (n=45, 32.61%), surgery rescheduled/canceled/already completed (n=23, 16.67%), change in method of appointment (n=17, 12.32%), and time conflict with another medical appointment (n=10, 7.25%) (Table 3).

**Table 3:** Patient-reported reasons for cancellations.

Reasons for Cancellation	n (%)
Patient request/reason	45 (32.61%)
Surgery rescheduled/cancelled/already completed	23 (16.67%)
Change of method of appointment	17 (12.32%)
Time conflict with another medical appointment	10 (7.25%)
Other	8 (5.8%)
System error	8 (5.8%)
Team request	8 (5.8%)
Appointment no longer needed	7 (5.07%)
Patient no show/late	6 (4.35%)
Appointment Rescheduled	3 (2.17%)
Technology Issue	3 (2.17%)

## Discussion

The COVID-19 pandemic prompted rapid implementation of telehealth visits in UCLA’s Anesthesiology and Perioperative Care Department. Using one of the largest preoperative anesthesia datasets of metropolitan, academic health systems, we analyzed evaluation completion using telemedicine compared to in-person consultation. 

Appointment completion, cancellation, no show

In alignment with previous literature, we found greater appointment completion and fewer cancellations in the telemedicine cohort [1,7-8,19]. This suggests that telemedicine has great potential to increase perioperative healthcare access to patients limited by geographical locations or socioeconomic status, which may be the reason for greater appointment adherence. We also found that the no-show rate in the telemedicine cohort was statistically similar to the in-person cohort. Previous studies have even demonstrated lower no-show rates in telemedicine groups [7,8,19]. Though telemedicine cannot fully replace in-person consultations, particularly given limitations in the physical exam, studies not only suggest that telemedicine improves access to quality healthcare but also that telemedicine visits can reduce medical costs for both patients and hospitals [18,27]. Future studies can also evaluate the quality of telemedicine visits in comparison to in-person visits from both patient and provider perspectives. 

Patient demographics

Patient demographics between telemedicine and in-person cohorts demonstrated differences in age, race, ethnicity, language, and insurance. These differences may elucidate potential shortcomings of the current state of telehealth. Demographically, this data demonstrates that patients who are older, who are of American Indian or Alaskan Native race, and Hispanic or Latino ethnicity were less likely to receive telemedicine appointments. Socioeconomically, patients who are non-privately insured, particularly those insured by Medicare, were less likely to receive telemedicine appointments. Lack of coverage or reimbursements from public insurance companies may be a reason for inequity. Furthermore, the lack of reimbursement parity between in-person and telemedicine consultations may widen the disparity. In fact, Kvedar and colleagues demonstrated substantial differences in reimbursement rates ($73.35 vs $94.05) in telemedicine and in-person respectively [27]. Furthermore, previous studies demonstrated a digital divide amongst those with lower annual incomes, older populations, and racial and ethnic minorities [1,18]. Overcoming digital divide inequalities may be a potential avenue to increase access to preoperative anesthesia telemedicine evaluations. 

Reasons for cancellation 

It is well established that a preoperative evaluation is necessary before proceeding with surgery. The preoperative visit serves as an opportunity to identify comorbidities and appropriately risk-stratify patients. Insufficient preoperative anesthesia assessment alongside patient medical comorbidities is one of the leading causes of surgical cancellation [7,8,16]. Delays in surgical care increase perioperative morbidity and mortality, economic costs for patients, and waste of hospital resources [7,8,16]. As such, it is important to understand the reasons for preoperative anesthesia appointment cancellation. The current study showed that the top four reasons for cancellation included patient request or reason, surgery rescheduled/canceled/already completed, change in method of appointment, and time conflict with another medical appointment.

Limitations

Although this is one of the largest analyses of preoperative anesthesia appointment cancellation in telemedicine and in-person consultation, there are several limitations that require attention. Most notably, given that this is a retrospective, non-randomized cohort study for telemedicine preoperative consultation, results can only demonstrate a strong association but not a causative relationship between telemedicine and higher appointment completion and lower cancellations. Although we compare appointment cancellations to in-person workflow, we did not randomize the patient population to test if telemedicine consultations were non-inferior to in-person consultations. At UCLA, the decision to use telemedicine or in-person for preoperative anesthesia consultations is generally determined by surgeon or anesthesiologist preference which can potentially introduce selection bias, a window for a potential risk stratification selecting low-risk procedures or patients based on comorbidities including medical or surgical history. Furthermore, as a retrospective study, we did not directly propensity score match the in-person and telemedicine cohorts to compare demographic and case cancellation data. To minimize type I error, the null hypothesis was only rejected when p-values < 0.05. Some of the relevant telemedicine issues that require further examination are the security of personal health information, patient selection procedures, and provider and patient satisfaction [10,28]. Future research could investigate the preference of telemedicine for preoperative anesthetic assessment now that COVID-19 quarantines and fears have been mitigated and an exploration of patient outcomes comparing in-person and telemedicine visits [10].

## Conclusions

This study demonstrates that telemedicine can improve preoperative anesthesia appointment completion and decrease appointment cancellations. During the COVID-19 pandemic, the use of telemedicine as an alternative to in-person evaluations was particularly relevant. We also demonstrate the potential shortcomings of telemedicine in serving patients who are older, require interpreter services, or are not privately insured. These disparities highlight potential avenues to increase health equity and access to telemedicine. Relevant telemedicine issues in the context of preoperative anesthesia evaluation that should be investigated further include the security of personal health information, patient selection procedures, and provider and patient satisfaction.
